# Ultrasensitive detection of SARS-CoV-2 RNA and antigen using single-molecule optofluidic chip

**DOI:** 10.1063/5.0049735

**Published:** 2021-06-01

**Authors:** G. G. Meena, A. M. Stambaugh, V. Ganjalizadeh, M. A. Stott, A. R. Hawkins, H. Schmidt

**Affiliations:** 1 School of Engineering, University of California, Santa Cruz, 1156 High Street, Santa Cruz, California 95064, USA; 2 Electrical and Computer Engineering Department, Brigham Young University, Provo, Utah 84602, USA

## Abstract

Nucleic acids and proteins are the two most important target types used in molecular diagnostics. In many instances, simultaneous sensitive and accurate detection of both biomarkers from the same sample would be desirable, but standard detection methods are highly optimized for one type and not cross-compatible. Here, we report the simultaneous multiplexed detection of SARS-CoV-2 RNAs and antigens with single molecule sensitivity. Both analytes are isolated and labeled using a single bead-based solid-phase extraction protocol, followed by fluorescence detection on a multi-channel optofluidic waveguide chip. Direct amplification-free detection of both biomarkers from nasopharyngeal swab samples is demonstrated with single molecule detection sensitivity, opening the door for ultrasensitive dual-target analysis in infectious disease diagnosis, oncology, and other applications.

## I. INTRODUCTION

There is a pressing demand for tools that simultaneously analyze multiple biomarker types, such as nucleic acids, proteins, and metabolites. This requirement is partially driven by the emergence of personalized medicine, single cell analysis, and the need to analyze a variety of genomic and proteomic biomarkers with high specificity and sensitivity—ideally at ultra-low concentrations for early disease detection.^1–5^ Another driver is the need to monitor different target types during different stages of a disease. One example is Zika virus (ZIKV) infection, in which RNAs are detectable early on (first 1–2 weeks) while protein biomarkers can be used for diagnosis many months post-infection.^6^ ZIKV presents particular challenges, as viremia is generally low and protein biomarkers exhibit cross-reactivity with Dengue infection.^7^ Thus, multi-target analysis would raise reliability and confidence. Another example is the acute respiratory illness Coronavirus disease 2019 (COVID-19), which is caused by severe acute respiratory syndrome coronavirus 2 (SARS-CoV-2), a highly infectious and harmful pathogen that attacks and destroys lung tissues.^8,9^ The disease has spread all over the world and, as of March 2021, already affected nearly 110 × 10^6^ people and caused over 2.5 × 10^6^ deaths. Reliable and sensitive diagnosis has proven to be the cornerstone of any strategy to combat the spread of the virus, and molecular biomarker detection is at the heart of current gold standard techniques. Both RT-PCR for viral RNA^10^ and enzyme linked immunosorbent assay (ELISA) and chemiluminescence immunoassays (CLIAs) for nucleocapsid antigens^11^ are used for COVID-19 confirmation. Each approach has its advantages (high sensitivity or speed), but neither is able to meet all desirable criteria. Therefore, a combination of rapid antigen testing with immediate confirmation of the result with nucleic acid-based would be highly desirable for early stage COVID-19 screening and quarantining^12,13^ when both target types are present. Unfortunately, the targets cannot currently be detected simultaneously due to the completely different underlying principles for each test.

Direct amplification-free detection of individual biomarkers has the potential to elegantly overcome these challenges and enable simultaneous diagnosis of different molecular target types with a single approach. Highly integrated lab-on-chip devices are attractive for this purpose due to their potential for point-of-care use, and several electrical and optical sensing modalities are being pursued for either target type.^14–19^ Optofluidic biosensors based on liquid-core (LC) anti-resonant-optical-waveguides (ARROWs) have emerged as a diagnostic platform for multiplex detection of individual biomolecules. Orthogonally intersecting solid-core (SC) waveguides for excitation and liquid-core (LC) waveguides for signal collection allow for optical detection of fluorescent targets in flow, which has enabled the detection of single Ebola RNAs with an ultrawide dynamic range.^20^ Multi-spot excitation patterns created by multi-mode interference (MMI) excitation waveguides were introduced for spectral,^21^ spatial,^22^ velocity-based,^23^ and combinatorial^24^ multiplexing of up to seven targets. In addition, detection of both nucleic acid and protein biomarkers for Zika viral infection was demonstrated on an MMI-based device.^25^ However, this demonstration was done with thousands of molecular targets bound to microbeads because of the challenge of creating bright enough fluorescent labels for individual antigens.

Here, we report the use of the ARROW photonic biosensor platform for the first dual detection of nucleic acid and antigen biomarkers with single molecule sensitivity from clinical SARS-CoV-2 nasopharyngeal (NP) swab samples. This is enabled by combining a bead-based extraction protocol, spatial–spectral multiplexing in a multi-channel chip, and novel bright fluorescent probes for protein targets in a single assay. We demonstrate amplification-free detection of both targets with high accuracy using efficient wavelet-based data analysis.

## II. DEVICE DESIGN AND EXPERIMENTAL SETUP

**FIG. 1.**
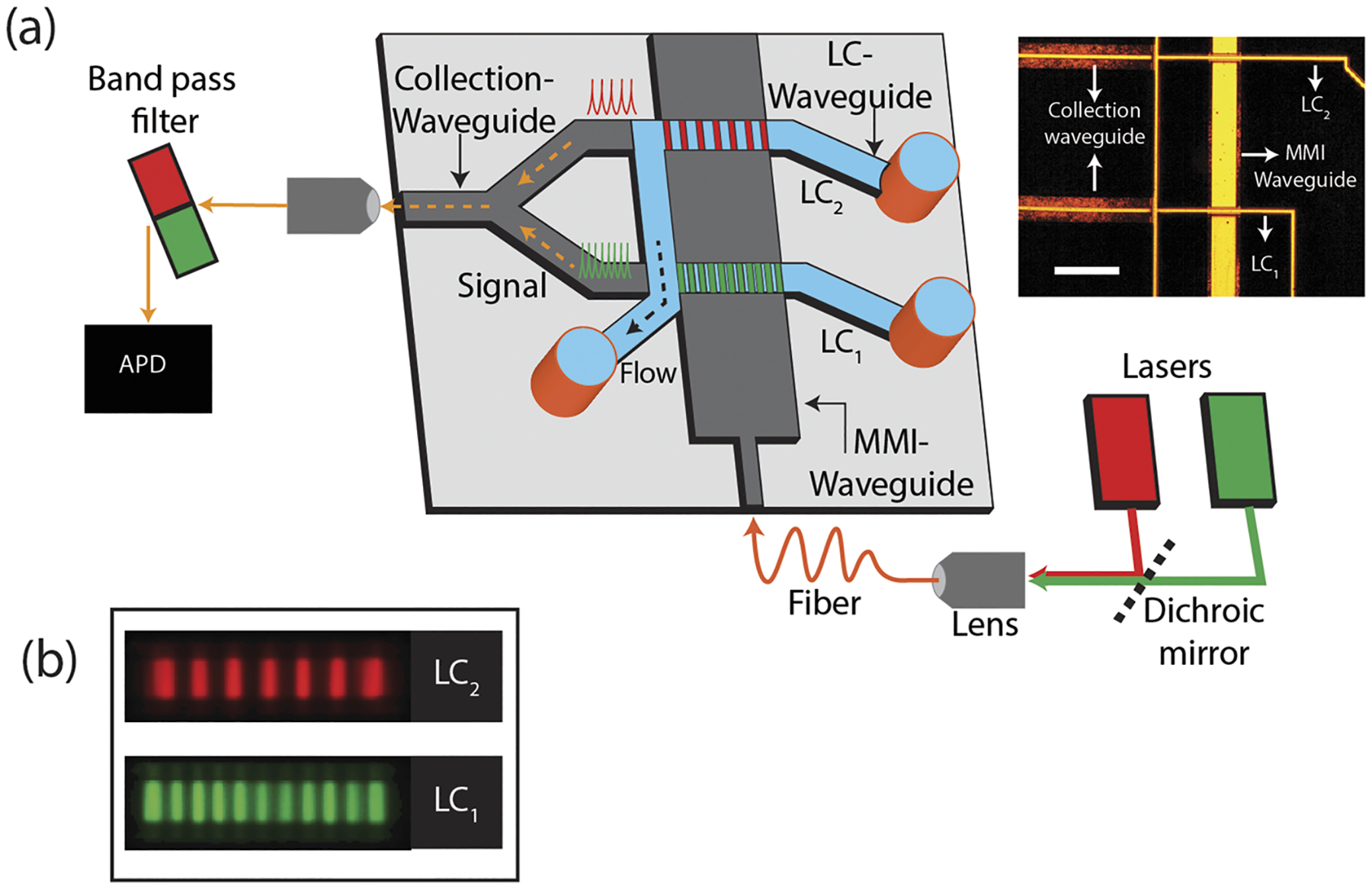
Device design: (a) cartoon of the ARROW biosensor chip and the experimental setup. The inset shows the top down optical image of the ARROW biosensor (scale: 200 *μ*m). (b) Top down color-coded fluorescence image of the excitation pattern generated by the MMI waveguide in LC_1_ and LC_2_, respectively.

Figure 1(a) shows the schematic diagram of the ARROW biosensor chip, which is fabricated using standard microfabrication techniques on top of a 100 mm silicon wafer. Six alternating layers of SiO_2_ and Ta_2_O_5_ dielectric thin films with thicknesses 265 and 102 nm, respectively, are sputtered to form the anti-resonant-reflecting layer stack that ensures low-loss light propagation in low-index materials. Two hollow microchannels of dimensions 12 × 6 *μ*m (width × height) are defined using a sacrificial SU-8 layer. A 6 *μ*m thick SiO_2_ layer is then deposited on the wafer using plasma-enhanced chemical vapor deposition. The MMI waveguide and collection waveguides are patterned using photolithography, and 3 *μ*m tall ridges are etched into the SiO_2_ layer via inductively coupled plasma reactive ion etching to form 3 *μ*m tall ridge SC waveguides. Finally, the sacrificial SU-8 is etched out in an acid bath and the resulting hollow channel can now be filled with a buffer medium to form the LC waveguides.^26^ The inset of Fig. 1(a) shows the top down optical image of the ARROW chip and how solid and liquid-core waveguides are interfaced for biosensing purposes. The planar single mode SC waveguide that expands into a wide MMI waveguide orthogonally intersects two LC channels. The MMI waveguide is wide enough to support multiple modes propagating at different propagation constants. These modes get excited by the single mode waveguide and interfere with each other constructively to produce self-images of the fundamental excitation mode. Thus, patterns with a number N_j_ of well-defined spots are generated along the MMI waveguide (effective width w_eff_) at certain lengths L_j_ for a specific wavelength *λ*_j_, as described by the following equation:

(1) $$N_{j}L_{j}=\frac{n_{c}w_{eff}^{2}}{\lambda_{j}}.$$

Here, an MMI waveguide of width 75 *μ*m is designed to intersect the first LC channels at length L_1_ = 1676 *μ*m to generate a pattern with 11 spots when excited at 556 nm wavelength and intersect the second LC channel at L_2_ = 2243 *μ*m to produce a pattern with seven spots at 633 nm wavelength. Figure 1(b) shows the top down images of the excitation spots produced in the fluorescent dye filled LC channels when excited by the MMI waveguide. The MMI waveguide is simultaneously coupled to both a He–Ne laser (633 nm) and a solid-state laser (556 nm). Fluorescently tagged targets flowing through the LC channels get excited by these MMI waveguide patterns and generate fluorescent signals corresponding to the respective wavelengths. The signals from the two LC waveguides are collected by two separate SC waveguides, which are combined into a single collection waveguide using a 2 × 1 *y*-splitter geometry for off-chip detection with an avalanche photodiode (APD). Based on the LC channel in which the target is flowing, the spatial and spectral information is encoded in the time-dependent fluorescence signal through the specific pattern produced by the MMI waveguide in the LC channels.

## III. BRIGHTLY FLUORESCENT RNA AND PROTEIN ASSAY PREPARATION

**FIG. 2.**
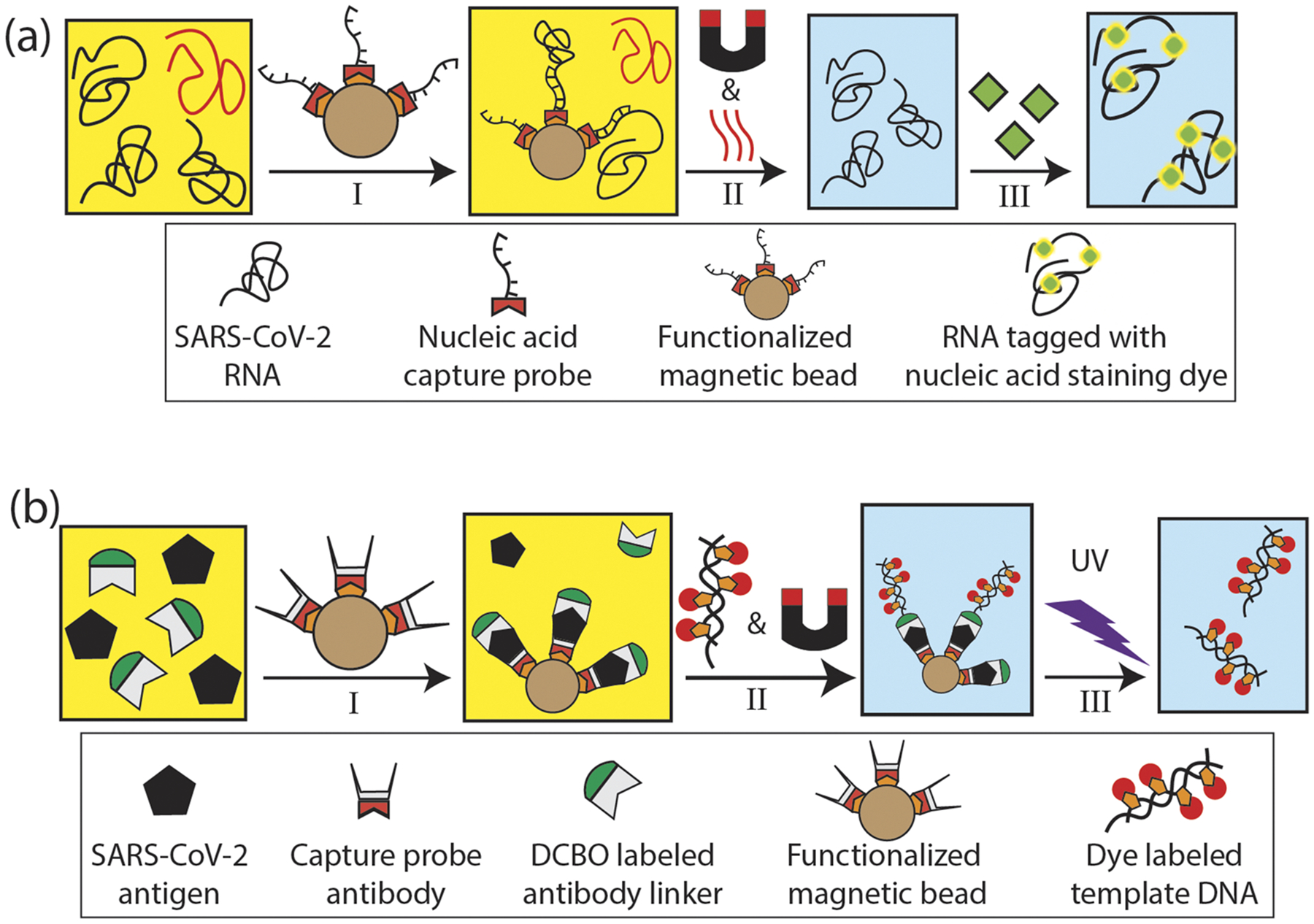
RNA and protein assay preparation steps: (a) nucleic acid assay preparation: I—capture of SARS-CoV-2 RNA targets spiked in NP swab using target specifically functionalized magnetic beads, II—unbound non-specific biomolecules and NP swab are washed off by buffer exchange using a magnet and the captured RNAs are thermally released and eluted, and III—RNA molecules are fluorescently tagged with nucleic acid staining dye. (b) Protein assay preparation: I—SARS-CoV-2 N antigen targets spiked in NP swab are tagged with DBCO labeled antibody and captured using functionalized magnetic beads, II—target antigens are tagged with fluorescently labeled template DNA reporter probes and unbound non-specific molecules are washed off, and III—captured reporters are cleaved off by UV exposure and eluted.

One key consideration for a practical dual-target assay for two very different molecular targets is the development of a common sample preparation protocol. Here, this is implemented with microbead-based extraction to achieve target specificity, followed by a labeling strategy that is optimized to provide single target sensitivity on the detection chip. The sample preparation steps are shown in Fig. 2. For nucleic acid detection [Fig. 2(a)], the region encoding the nucleocapsid (N) protein is used to target specifically capture and separate the SARS-CoV-2 RNA from cell lysate (BEI resource: NR-52286, heat inoculated cell lysate of Vero E6 cells infected with SARS-CoV-2). The lysate was mixed with RNA shield (Zymo research) in one to one volume ratio and spiked in negative nasopharyngeal (NP) swab samples from the UCSC Molecular Diagnostics Lab (also mixed with RNA shield) to give a final stock RNA concentration of 3 × 10^8^ genomic copies/ml. SARS-CoV-2 RNA was specifically captured on streptavidin coated magnetic beads functionalized with biotinylated capture oligonucleotides specific to the N protein region (MT246667.1, nt 28 286–28 340). 30 *μ*l of this sample was heated to 80 ^○^C for 5 min to uncoil the RNA. 2 *μ*l of the functionalized magnetic beads (4 × 10^9^ beads/ml) was added, and the mixture was incubated for more than an hour. The beads with the RNA were pulled down with a magnet, and all unbound targets, cell lysate, and NP swab were washed off by five to six buffer exchanges that use buffer volume three times more than the sample volume to prevent any contamination. The beads were resuspended in 10 *μ*l of nuclease free and RNase free buffer and heated to 80 °C to release the captured RNA, which was eluted and tagged with 1 *μ*M of green POPO-3 nucleic acid staining dye.

The protein assay, illustrated in Fig. 2(b), is also based on functionalized microbeads to capture the molecular target, here the SARS-CoV-2 nucleocapsid (N) antigen. Bead pulldown and labeling was facilitated with target specific antibodies. Specifically, 5 *μ*l of magnetic beads (9 × 10^9^ beads/ml) were incubated with 5 *μ*g (≈5× molar excess) of biotinylated capture SARS-CoV-2 N protein antibody (HM1054, East Coast Bio). After washing off unbound antibodies, the magnetic beads with the capture antibody were subtended in 5 *μ*l 1 × PBS buffer. 2 *μ*g (≈5× molar excess) of a secondary, DBCO labeled anti-SARS-CoV-2 N protein antibody (HM1055, East Coast Bio.) and 1 *μ*g of target (SARS-CoV-2 N protein model, East Coast Bio.) antigens spiked in 50 *μ*l of NP swab were added to the bead-pulldown complex and incubated at 37 °C for 2 h.

Importantly, in order to create a probe that is bright enough for single protein detection, we developed a novel reporter based on modified template DNA with biotinylated-dUPT, tagged with multiple cyanine dyes. Briefly, 1 kbp of pUC-19 PCR product was made with photocleavable azide forward primer (IDT) and 50% biotinylated-dUTP. To act as a fluorescence probe, the DNA was then functionalized with Cy-5 (labeled monovalent streptavidin—mSA, Howarth Lab, Oxford University) so that there are ~250 mSA/1 kbp and up to 750 dyes/probe.

30 *μ*l of about 100 nM concentration of this reporter probe was added to the antigen-spiked swab samples and incubated. The photocleavable azide of the reporter probe reacts and hybridizes with the DBCO-labeled antibody–antigen complex on the magnetic bead thus effectively tagging each of the captured antigens. This capture assay was washed to remove the NP swab and any unbound reporter probes and resuspended in 50 *μ*l of 1 × PBS. An aliquot of this (5 *μ*l) was subjected to UV-B (311 nm) light for 45 s to cleave the reporter probes from the bead complex at the photocleavable azide junction. The beads were pulled down with a magnet, and the cleaved reporter probes were eluted and diluted (1:10). Assuming 100% capture and labeling efficiency by the beads, this corresponds to a nominal concentration of 6.4 ng/ml of target antigen. Detection of the eluted bright fluorescent reporters is then fully equivalent to sensing the individual target antigens.

## IV. AMPLIFICATION-FREE DETECTION OF SINGLE RNA AND ANTIGEN TARGETS

**FIG. 3.**
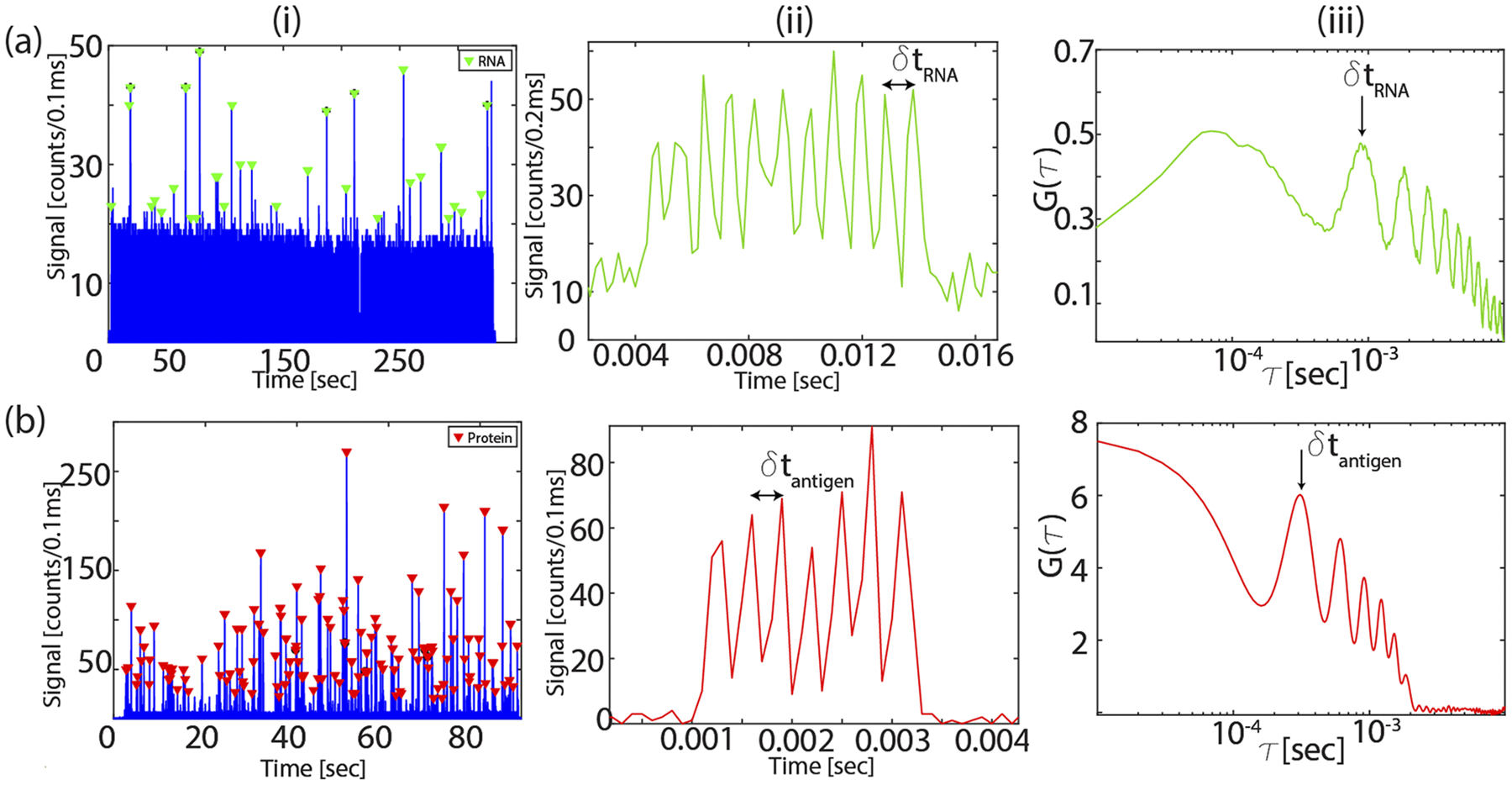
Fluorescence signal: (a) single RNA detection: i—fluorescence signals from RNA molecules flowed through LC_1_ and excited by the MMI waveguide coupled to 556 nm laser, ii—zoomed-in signal from a single RNA with 11 peaks, and iii—corresponding single RNA autocorrelation signal. The peak number depends on the excitation pattern and gives the characteristic time δt of the target. (b) Same analysis for single antigen target detection using the seven-spot pattern in LC_2_.

**FIG. 4.**
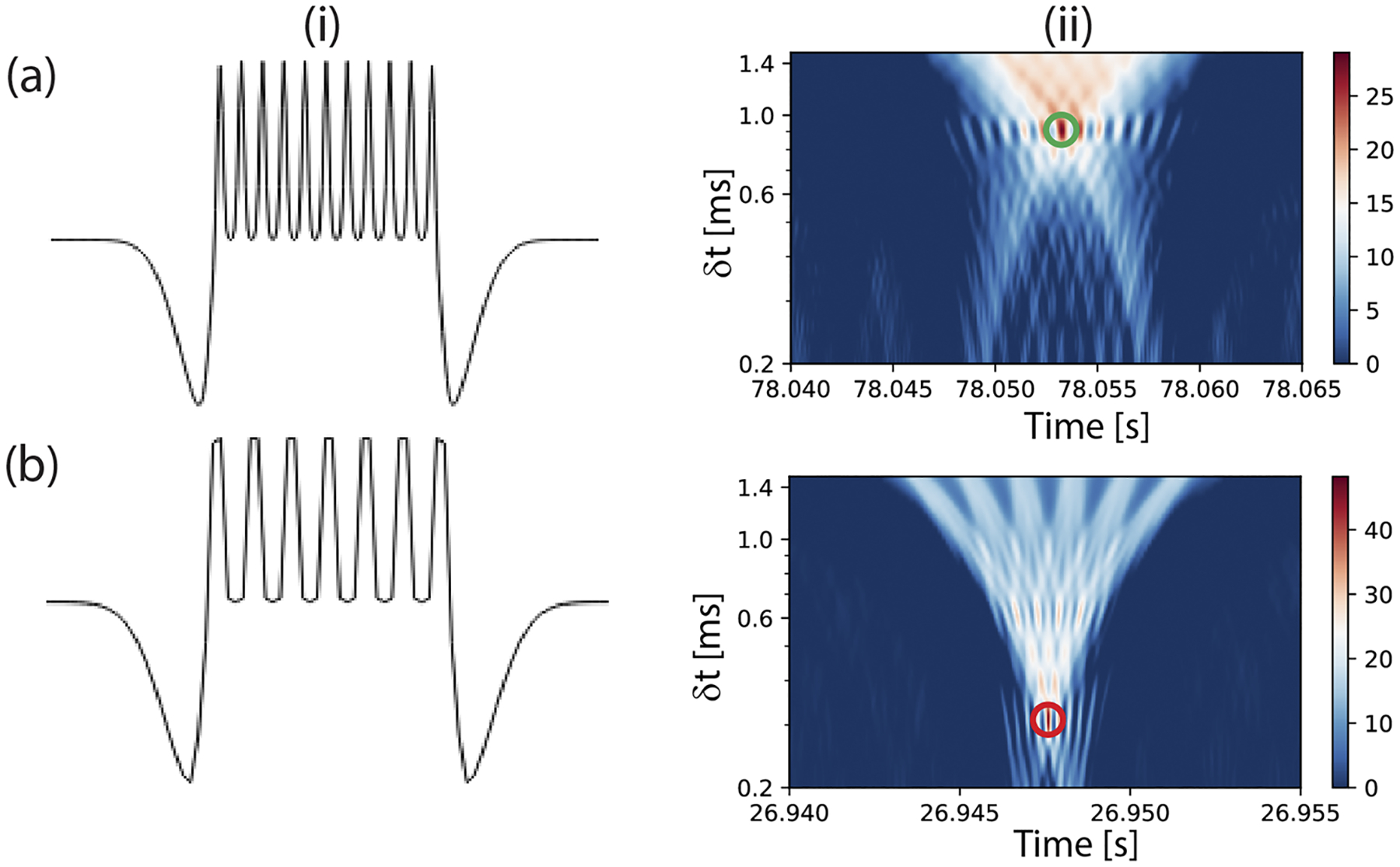
CWT analysis: (a) CWT analysis for RNA signal processing: i—custom wavelet function with 11 Gaussian peaks margined with negatively skewed peaks to analyze fluorescence signals from RNA targets and ii—example of a 2D map of the CWT coefficients [Eq. (3)] obtained when a signal from the RNA target [Fig. 3(a–iii)] is transformed with the corresponding wavelet function. The green circle indicates the presence of a local maximum. (b) The wavelet function for analyzing signals from proteins and an example of the CWT coefficients from processing an event [Fig. 3(b–iii)].

As a first step toward a fully multiplexed dual-target assay, we demonstrate the ability to separately detect fluorescently labeled SARS-CoV-2 RNA molecules and the antigen reporters with single molecule sensitivity using the ARROW biosensor. Accordingly, green fluorescently tagged RNA molecules (Sec. III) are flowed through LC_1_ and buffer through LC_2_ and excited at 556 nm using the MMI waveguide. Figure 3(a–i) shows the fluorescence signals from individual RNA molecules detected by the APD as they flow past the MMI waveguide excitation region. A burst of photon counts above the background corresponds to a signal from an individual RNA. Figure 3(a–ii) shows a zoomed-in view of one such event. The fluorescence signal has 11 peaks corresponding to the excitation pattern generated by the MMI waveguide in LC_1_. *δ*t, the time between two adjacent peaks of the signal, is the characteristic lag time the molecule takes to flow past two adjacent excitation spots. One way to reliably and accurately obtain *δ*t is using autocorrelation. Figure 3(a–iii) shows the autocorrelation function G(*τ*) of the signal, given by the following equation:

(2) $$G(\tau )=\frac{\langle f(t)\cdot f(t+\tau )\rangle }{{\langle f(t)\rangle }^{2}},$$

where f(t) is the time domain fluorescence signal and the brackets represent integration over the entire duration of the event. In typical fluorescence correlation spectroscopy experiment with single excitation spots, the resulting autocorrelation curves show a monotonous decay with increasing *τ* due to drift and diffusion of the particles out of the excitation volume.^27^ In our case, because the MMI waveguides produce an excitation pattern with multiple spots that is reproduced in the fluorescence signal, G(*τ*) has a number of distinct peaks at integer multiples of the characteristic lag time *δ*t that correspond to the time it takes the particle to travel between adjacent excitation spots. In a second experiment, the antigen reporters (Sec. III) were flowed through LC_2_ (buffer in LC_1_) with the MMI waveguide coupled to a 633 nm laser. Negative control experiments were also done with the assay prepared using Zika RNA and Zika NS1 antigen targets spiked in NP swab. No false negative signals were observed, which confirms the excellent specificity of the bead-based target extraction protocol. Zooming into one of the events from the reporters in Fig. 3(b–i) shows a fluorescence signal from an individual bright reporter with seven peaks [Fig. 3(b–ii)]. Figure 3(b–iii)) shows the corresponding autocorrelation with six peaks. The *δ*t values along with the dimensions of the waveguide give the flow velocity of the targets. The molecules flowing past the excitation region have a broad distribution in the velocity and the cross-sectional position with respect to the MMI waveguide.^28^ This results in variations in the signal intensity and the temporal width, *δ*t, of the signals. For efficient signal detection and identification, which takes this into account, the fluorescence trace is processed using continuous-wavelet-transform (CWT) analysis,

(3) $$C(t,\delta t)=\langle f,\psi_{t,\delta t}\rangle =\int_{-\infty }^{+\infty }f\left(t^{\prime }\right)\frac{1}{\sqrt{\delta t}}\psi^{*}\left(\frac{t^{\prime }-t}{\delta t}\right)dt^{\prime },$$

where f(t) is the time domain fluorescence signal trace and ψ(t, δt) is the wavelet function dependent on the time variable “t” and a scaling factor, δt >0, corresponding to the characteristic time of the target molecules. The fluorescence signal is correlated with the wavelet function ψ(t) [Eq. (3)]. The wavelet function is simultaneously translated (using parameter t) and dilated (using parameter δt) in the time domain to give the correlated CWT coefficients C(t,δt), visualized in a 2D map. The δt stretches and compresses the wavelet function to give the highest correlation with the multipeak time domain fluorescence signal. Here, for accurate correlation with the fluorescence signal of 11 peaks from the RNA molecules, a matched wavelet function [Fig. 4(a–i)] comprised of a sum of 11 Gaussians scaled and separated by the parameter “δt” and margined with negatively skewed peaks is used. For analyzing fluorescence signals from the antigen reporter, a wavelet function with seven peaks is used [Fig. 4(b–i)].

The fluorescence trace of each target is processed with both wavelet functions. Figure 4(a–ii) shows an example of the 2D CWT coefficient map when a signal from an RNA molecule is correlated with the wavelet function with 11 peaks. The signal is detected by the presence of a local maximum in the 2D map (depicted by the green circle). The same signal is also analyzed with a wavelet function with seven peaks. The event is identified as an RNA signal because the CWT coefficient value is higher when analyzed using the wavelet function with 11 peaks compared to using the seven peak wavelet function. Figure 4(b–ii) shows a similar 2D map of the CWT coefficients from an antigen reporter signal when processed with the corresponding wavelet function. Analyzing the fluorescence trace from the experiment showed events detected at an average rate of 1.9 events/s. This confirms rapid detection and identification of targets with single molecule sensitivity.

## V. MULTIPLEXED AMPLIFICATION-FREE DETECTION OF SARS-CoV-2 RNA AND ANTIGENS

**FIG. 5.**
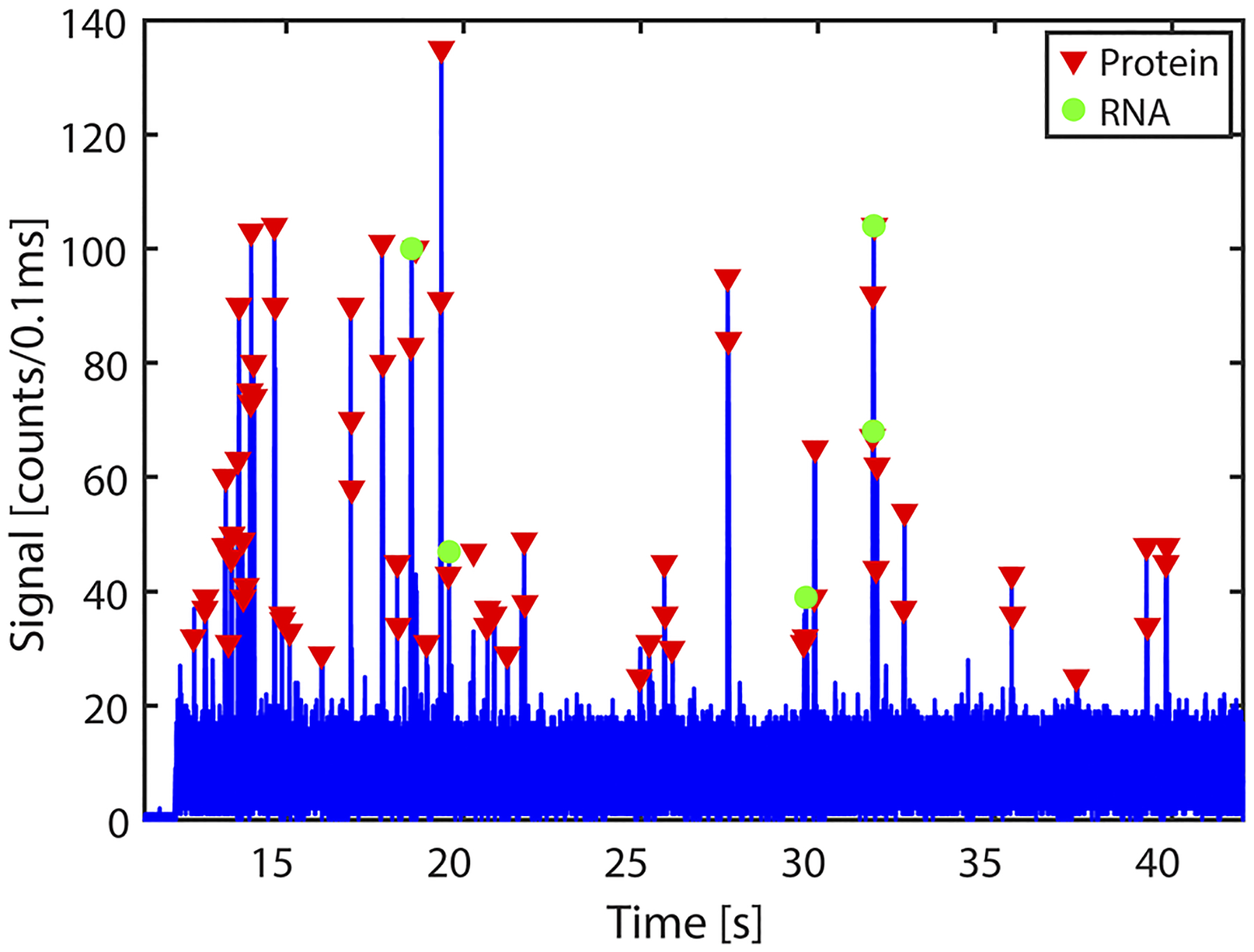
RNA and protein multiplexed detection: fluorescence signals from RNA and proteins from LC1 and LC2, detected simultaneously when the MMI is excited with both lasers and identified using the CWT analysis.

For dual detection of nucleic acid and protein targets, fluorescently tagged RNA molecules and the antigen reporters are flowed simultaneously in LC_1_ and LC_2_, respectively, and the MMI waveguide is excited by both the red and green lasers. To test the capability of the chip to simultaneously detect clinically relevant concentration of SARS-CoV-2 RNA and protein, starting concentrations of about 3 × 10^8^ genomic copies/ml (RNA) and 6.4 ng/ml (antigen) were used for the experiment. These lie within the concentration range of SARS-CoV-2 clinical samples during the early stage of infection.^29,30^ The signals from the two LC channels are collected by the 2 × 1 collection SC waveguides and sent to the APD. Signals corresponding to individual RNA and protein molecules with 11 and seven peaks, respectively, are detected and identified with the CWT analysis using the two custom wavelet functions. Figure 5 shows the fluorescence trace with both RNA and antigen reporters detected simultaneously. RNA and antigen reporter molecules were detected at a rapid rate of 0.7 events/s and 2 events/s, respectively, with an average signal strength of 48(±23) counts/100 *μ*s. The events were detected by the sensor with an average signal-to-noise ratio (SNR) of 8.1 showing very high sensitivity (SNR—defined as the ratio of the average signal strength to the average background noise; the average photon count from experiments done with blank samples is used as background noise—5.9 counts/100 *μ*s). This demonstrates, for the first time, multiplexed amplification-free detection of SARS-CoV-2 nucleic acids and antigens in a single assay. The high sensitivity of the device enables direct counting of targets at clinically relevant target concentrations. The limits of detection were assessed independently. We have previously demonstrated that the nucleic acid assay has a limit of detection in the attomolar range.^20,31^ For the antigen assay, the limit of detection was determined using serial dilution of the SARS-CoV-2 antigens and found an LoD of 0.7 ng/ml. This can be further improved by optimizing the assay (e.g., by enhancing the mixing process of targets and capture beads^31^). Additional ways to maximize the number of detected targets include on-chip integration of the sample preparation steps^20^ and increasing the fluorescence signal strength, e.g., by increasing the brightness of the fluorescent reporters, hydrodynamic focusing of the targets to the channel center,^32^ or using a liquid with higher refractive index in the detection channel.^33^

## VI. CONCLUSION

We have demonstrated an optofluidic biosensor for amplification-free, multiplexed dual detection of SARS-CoV-2 nucleic acids and proteins from clinical NP swab samples. Simultaneous detection of both targets was achieved by spectral and spatial multiplexing with an MMI waveguide integrated with two LC waveguides. An innovative bright fluorescent reporter was developed to enable single antigen detection, and CWT analysis with custom wavelet functions was used to identify and demultiplex the time domain fluorescence signals of both targets. This demonstration points the way toward ultrasensitive assays for multiplexed detection of different molecular biomarker types with applications in infectious disease diagnosis, oncology research, companion therapy, and more. In the future, the integration level and the detection efficiency of the chip-based sensor can be further improved by incorporating sample preparation^34^ and a laser source on the chip.^35^

## Data Availability

The data that support the findings of this study are available from the corresponding author upon reasonable request.
